# Amphibian skin-associated *Pigmentiphaga*: Genome sequence and occurrence across geography and hosts

**DOI:** 10.1371/journal.pone.0223747

**Published:** 2019-10-11

**Authors:** Molly C. Bletz, Boyke Bunk, Cathrin Spröer, Peter Biwer, Silke Reiter, Falitiana C. E. Rabemananjara, Stefan Schulz, Jörg Overmann, Miguel Vences

**Affiliations:** 1 Department of Biology, University of Massachusetts Boston, Boston, MA, United States of America; 2 Zoological Institute, Technische Universitt Braunschweig, Braunschweig, Germany; 3 DSMZ, German Collection of Microorganisms and Cell Cultures, Braunschweig, Germany; 4 Institute of Organic Chemistry, Technische Universität Braunschweig, Braunschweig, Germany; 5 Institute for Insect Biotechnology, Justus Liebig University Giessen, Giessen, Germany; 6 Department of Zoology and Animal Biodiversity, University of Antananarivo, Antananarivo, Madagascar; 7 Microbiology Institute, Technische Universität Braunschweig, Braunschweig, Germany; Sichuan University, CHINA

## Abstract

The bacterial communities colonizing amphibian skin have been intensively studied due to their interactions with pathogenic chytrid fungi that are causing drastic amphibian population declines. Bacteria of the family *Alcaligenaceae*, and more specifically of the genus *Pigmentiphaga*, have been found to be associated specifically to arboreal frogs. Here we analyze their occurrence in a previously assembled global skin microbiome dataset from 205 amphibian species. *Pigmentiphaga* made up about 5% of the total number of reads in this global dataset. They were mostly found in unrelated arboreal frogs from Madagascar (Mantellidae and Hyperoliidae), but also occurred at low abundances on Neotropical frogs. Based on their 16S sequences, most of the sequences belong to a clade within *Pigmentiphaga* not assignable to any type strains of the five described species of the genus. One isolate from Madagascar clustered with *Pigmentiphaga aceris* (>99% sequence similarity on 16S rRNA gene level). Here, we report the full genome sequence of this bacterium which, based on 16S sequences of >97% similarity, has previously been found on human skin, floral nectar, tree sap, stream sediment and soil. Its genome consists of a single circular chromosome with 6,165,255 bp, 5,300 predicted coding sequences, 57 tRNA genes, and three rRNA operons. In comparison with other known *Pigmentiphaga* genomes it encodes a higher number of genes associated with environmental information processing and cellular processes. Furthermore, it has a biosynthetic gene cluster for a nonribosomal peptide syntethase, and bacteriocin biosynthetic genes can be found, but clusters for β-lactones present in other comparative *Pigmentiphaga* genomes are lacking.

## Introduction

The cutaneous microbiome of amphibians has become a well-studied system, triggered by the rise of the pathogenic fungi, *Batrachochytrium dendrobatidis* (*Bd*) and *B*. *salamandrivorans* (*Bsal*). These fungi colonize the amphibian skin and are causing drastic population declines and extinctions in this class of animals [1,2]. The bacterial communities associated to amphibians interact with these fungi and some of these bacteria have the potential to inhibit the growth of *Bd* and *Bsal*, thus providing protection to their hosts [3].

Recent research based on next-generation amplicon sequencing of the 16S rRNA gene show that bacterial communities on the skin of amphibians are predominantly composed of common bacteria recruited from environmental reservoirs [4], and their dominant members can be readily cultured [5]. Unsurprisingly these communities are strongly controlled by environmental factors, e.g., bioclimate [6] and microhabitat [7–9]. However, clear differences have also been found between co-occurring hosts [7,10,11], suggesting that the skin mucosal differences among amphibian species act as filters determining which bacterial species are recruited into the community.

Considering the strong environmental influences on the amphibian cutaneous microbiome, it is of particular interest to analyze in more depth those bacteria that regularly colonize this habitat but are restricted to certain host taxa or host ecomorphs. An in-depth understanding of the genomic background, variation, phylogenetic relationships, and distribution of these bacteria may offer clues to understand which traits predispose them to successfully colonize this particular habitat.

This study was triggered by the observation that operational taxonomic units (OTUs) of the family *Alcaligenaceae* were strongly associated to arboreal ecomorphs in a study on Madagascan amphibians [12], and were also found to be a common member of the cutaneous microbiome of several Central American tree frogs, such as *Agalychnis callidryas* [13,14]. The family was represented by a pure culture isolate identified as *Pigmentiphaga* by 16S sequences in our bacterial culture collection from Madagascar frog skin [15], and also the sequences of *Alcaligenaceae* OTUs identified by amplicon sequences from Madagascar frogs.

*Pigmentiphaga* is a genus of the family *Alcaligenaceae*, assigned to the order Burkholderiales order within the Betaproteobacteria, and currently containing five species: the type species, *P*. *kullae*, plus *P*. *aceris*, *P*. *daeguensis*, *P*. *litoralis*, and *P*. *solis* [16–20]. According to these descriptions, the genus contains gram-negative, facultatively anaerobic, motile or nonmotile, catalase- and oxidase-positive, rod-shaped bacteria, found in diverse environments: *P*. *daeguensis* from dye wastewater, *P*. *litoralis* from tidal sediment, *P*. *soli* from soil, *P*, *aceris* from tree sap [17–20], and an unidentified species from tree-associated nematodes [21]. Furthermore, *Pigmentiphaga* have also been isolated from human clinical material [22], and genome sequences are available from these isolates [23]. Pigmentiphaga have been studied in the context of their ability to degrade azo dyes and aniline [16,24], and their role also has been discussed in the context of biphenyl-degradation [25].

Here, we analyze the occurrence of OTUs assigned to *Pigmentiphaga* on the skin of amphibians across taxa, geography and ecomorphs, assemble the full genome sequence of one *Pigmentiphaga* isolate obtained from a Madagascan frog, and analyze the phylogenetic relationships and differentiation of this isolate.

## Methods

### Analysis of amplicon data

To explore the distribution of the focal bacterial groups on amphibian hosts we used a recently published global dataset of amphibian skin microbiomes [6], and extracted all OTUs assigned to the family *Alcaligenaceae* and genus *Pigmentiphaga*. This global dataset is a compilation of targeted amplicon sequencing data of the 16S rRNA gene (V4 region) generated on Illumina sequencing platforms. It contains skin microbiome samples from 2,349 post-metamorphic amphibians, comprising 27 amphibian families (205 species) collected from 12 countries (5 continents) [6]. In this study, quality-filtered Illumina reads were classified into sub-operational taxonomic units (sOTUs) using the deblur pipeline [26]. These data were subsequently rarified and taxonomy was assigned using the Ribosomal Database Classifier [27] in QIIME [28] (see [6] for detailed methods). We examined the distribution of the genus *Pigmentiphaga* across locations (i.e. countries), host species, and host ecomorphological classes. To explore the distribution of the focal bacteria across host phylogeny, a phylogenetic tree of arboreal and scansorial host genera was built using timetree.org [29]. All plots were produced with ggplot2 [30].

### Isolate sampling

The bacterium for which we here report the full genome sequence was isolated on 1% tryptone agar from a skin swab of a single individual of the amphibian species, *Mantella crocea* at Torotorofotsy "Prolemur Camp" near Andasibe, Madagascar (-18.7709 S, 48.43222 E). This individual frog was captured by gloved hands and kept in a sterile Whirl-Pak bag for no longer than 1 hour before sampling. Frog skin microbiota were sampled by first rinsing the skin to remove transient microbes and then swabbing the ventral skin with a sterile rayon tipped swab 10 times. Swabs were stored in Tryptic Soy Yeast Extract media with 20% Glycerol and frozen until processing. The frog was immediately release at the site of capture after sampling.

### DNA extraction and complete genome sequencing

DNA was isolated using Qiagen Genomic-tip 100/G (Qiagen, Hilden Germany) according to manufacturer instructions. SMRTbell™ template library was prepared according to the instructions from PacificBiosciences, Menlo Park, CA, USA, following the Procedure & Checklist–Greater Than 10 kb Template Preparation. Briefly, for preparation of 15kb libraries 8μg genomic DNA was sheared using g-tubes™ from Covaris, Woburn, MA, USA according to the instructions of the manufacturer. DNA was end-repaired and ligated overnight to hairpin adapters applying components from the DNA/Polymerase Binding Kit P6 from Pacific Biosciences, Menlo Park, CA, USA. Reactions were carried out according to the manufacturer instructions. BluePippin™ Size-Selection to greater than 4 kb was performed according to the manufacturer's instructions (Sage Science, Beverly, MA, USA). Conditions for annealing of sequencing primers and binding of polymerase to purified SMRTbell™ template were assessed with the Calculator in RS Remote, PacificBiosciences, Menlo Park, CA, USA. 1 SMRT cell was sequenced on the PacBio *RSII* (PacificBiosciences, Menlo Park, CA, USA) taking one 240-minutes movie. SMRT sequencing revealed a total number of 85,590 reads with a mean read length of 12,138 bp and a N50 value of 16,449 bp. From the same batches of DNA, short insert libraries were created using the Illumina Nextera XT DNA Library Prep Kit (Illumina, San Diego, CA, USA) and sequenced on an Illumina MiSeq (Illumina, San Diego, CA, USA) resulting in 7,916,070 paired-end reads of 2x76 bp.

### Genome assembly and annotation

Genome assembly was performed applying the RS_HGAP_Assembly.3 protocol included in SMRT Portal version 2.3.0 using default parameters. The assembly revealed a single circular chromosome with a coverage of 133x. The chromosome was circularized, artificial redundancies at the ends of the contigs were removed and adjusted to *dnaA* as the first gene. Error-correction was performed by a mapping of Illumina short reads onto finished genome using Burrows-Wheeler Alignment bwa 0.6.2 in paired-end (sample) mode using default setting [31] with subsequent variant and consensus calling using VarScan 2.3.6 (Parameters: mpileup2cns—min-coverage 10—min-reads2 6—min-avg-qual 20—min-var-freq 0.8—min-freq-for-hom 0.75—p-value 0.01—strand-filter 1—variants 1—output-vcf 1) [32]. A consensus concordance of QV60 could be reached. Automated genome annotation was carried out using Prokka [33] and NCBI PGAP [34].

### Genome comparisons

We used Mauve software [35] to compare gene arrangement of the newly sequenced genome with those of other available *Pigmentiphaga* genomes. We compared the general gene functional characterization (KEGG functional categories) of the newly sequenced genomes with all other available *Pigmentiphaga* genomes using BlastKOALA [36]. On average, 50% of genomes’ protein coding sequences were annotated. Identification of natural product gene clusters was performed with the antibiotics and Secondary Metabolite Analysis SHell (antiSMASH version beta5; https://antismash.secondarymetabolites.org) [37] AntiSMASH is an online platform that allows for a genome-wide identification and analysis of secondary metabolite BGCs in bacterial genomes, by integrating and cross-linking with a large number of *in silico* secondary metabolite analysis tools like CLUSEAN [38], BAGEL2 [39], ClustScan [40], and NORINE [41].

### Phylogenetic analysis

We performed BLAST searches against the NCBI database, using the full 16S rRNA gene sequence from the sequenced genome to understand the distribution of this bacterium outside of amphibian hosts. We also used the MOLE-BLAST tool of NCBI to retrieve from GenBank the sequences of bacterial taxa most closely related to the *Alcaligenaceae* sOTUs in our amplicon data set. MOLE-BLAST is an experimental tool to find closest database neighbors of submitted query sequences, by computing a multiple sequence alignment (MSA) between the query sequences along with their top BLAST database hits.

The obtained sequences, along with sequences of our isolates and amplicon-derived sOTU sequences, were aligned with the MAFFT 7.0 algorithm [42] and phylogenetically analyzed in MEGA v. 7 [43]. We successively filtered the data set to remove highly deviant sequences retrieved from the database (as well as sOTU sequences associated to them), as identified by obvious alignment artefacts and excessively long branches in exploratory phylogenetic trees. The final Maximum Likelihood (ML) tree was computed under the GTR+G model of sequence evolution as determined under the Bayesian Information criterion in MEGA 7.

### Volatile compound analysis

A bacterial culture of the target bacterial strain was incubated on 1% tryptone agar for seven days at room temperature. Headspace extracts were obtained using a vacuum pump to draw clean air (purification by active charcoal filter) through a 250 mL glass vessel containing the culture plate. The air was then passed through a thermal desorption tube filled with an absorbent (Tenax TA Tube; GERSTEL, Mülheim an der Ruhr, Germany) for 5 h (three replicates).

Thermal desorption tubes were desorbed using a thermal desorption unit (TDU), cooled injection system (CIS) and a MultiPurposeSampler (MPS) autosampler (GERSTEL, Mülheim an der Ruhr, Germany) connected to an Agilent 7890B gas chromatograph. The gas chromatograph was equipped with a HP-5 MS fused silica capillary column (30 m, 0.25 i. d., 0.25 μm film, Hewlett-Packard, Wilmington, USA) connected to an Agilent 5977A mass-selective detector. Conditions: transfer line 300°C, electron energy 70 eV. Thermal desorption: 30°C, increasing at 60°C/min to 280°C (10 min isothermal). Cooled injection: -150°C, increasing at 12°C/min to 300°C (3 min isothermal). Gas chromatographic method: 50°C (5 min isothermal), increasing at 5°C/min to 320°C, and operated in splitless mode. Helium was used as carrier gas at 1.2 ml/min. GC retention indices (*RI*) were determined from a homologous series of *n*-alkanes (C_8_-C_30_). Compounds were identified by comparison of mass spectra and retention indices with those of authentic samples.

## Results and discussion

### Representation of *Alcaligenaceae* in amphibian cutaneous microbiomes

In the global dataset of amplicon sequences from 205 amphibian species [6] *Alcaligenaceae* sOTUs made up 284,771 out of 5,872,500 total rarified reads in the final data set (4.8%). *Alcaligenaceae* sOTUs (at a minimum threshold of 5 reads) were found in a total of 119 amphibian species from eight countries, and *Pigmentiphaga* sOTUs in 95 amphibian species. In a culture database of amphibian skin bacteria [22, Bletz & Woodhams unpublished data] 28 of 5938 isolates were from the *Alcaligenaceae*, and our isolate was the sole member from the genus *Pigmentiphaga*. Therefore, apparently, *Alcaligenaceae* and more specifically *Pigmentiphaga* appear to be underrepresented in culture databases of amphibian skin microbiota and thus might be less readily culturable than other bacteria from this habitat [5].

The family *Alcaligenaceae* currently contains 27 genera (UniProt 2019); in the amphibian microbiome data set, 338 out of a total of 124,348 sOTUs were assigned to this family, and of these, 35 to the genus *Pigmentiphaga* (reads = 266,723). The remaining *Alcaligenaceae* reads were assigned to the genera *Achromobacter* (n = 3,355), *Alcaligenes* (n = 666), *Sutterella* (n = 20) *Oligella* (n = 16), *Denitrobacter* (n = 3), *Candidimonas* (n = 12) or were left unassigned to a specific genus (n = 13,976).

**Fig 1:**
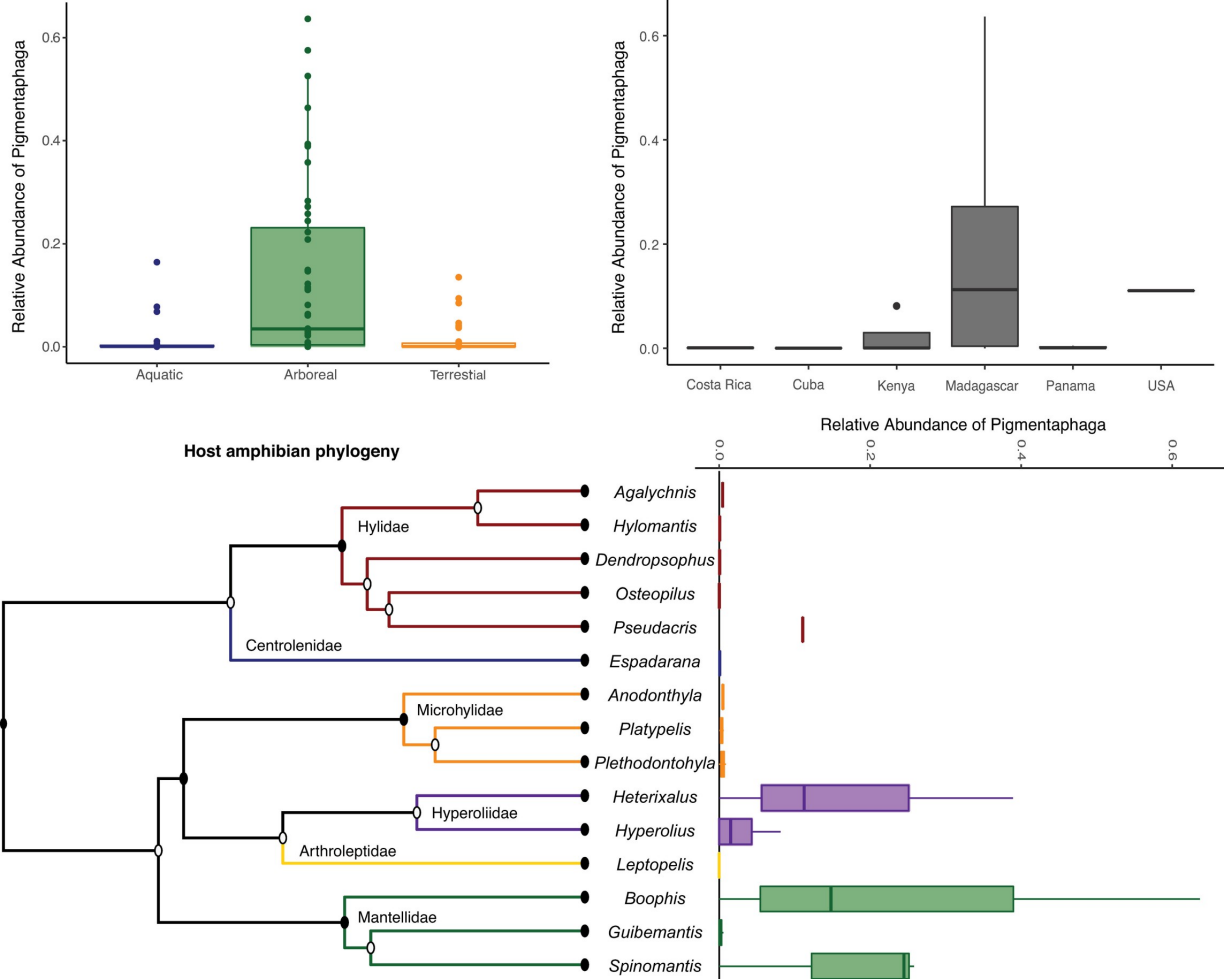
Distribution of *Pigmentiphaga* spp. across amphibian hosts. relative abundance within amphibian skin microbiomes across host eco-morphology classes (A), countries (B), and phylogeny of arboreal and scansorial amphibian hosts (C).

Confirming previous findings [12–14], in our global skin microbiome dataset [6] *Pigmentiphaga* was predominantly found on arboreal species as well as scansorial hosts within the amphibian clades Mantellidae (mean: 7.7% + 1.5%SD) and Hyperoliidae (mean: 7.9% + 4.7% SD, which are distributed in Madagascar, and (Hyperoliidae only) in mainland Africa (Fig 1). The genus also appears on amphibians from the genus *Pseudacris* (Fig 1). Overall, *Pigmentiphaga* was more common on amphibians from Madagascar; however, this could be associated with extensive sampling of arboreal hosts within this country.

### Phylogenetic diversity of *Pigmentiphaga* in amphibian cutaneous microbiomes

**Fig 2:**
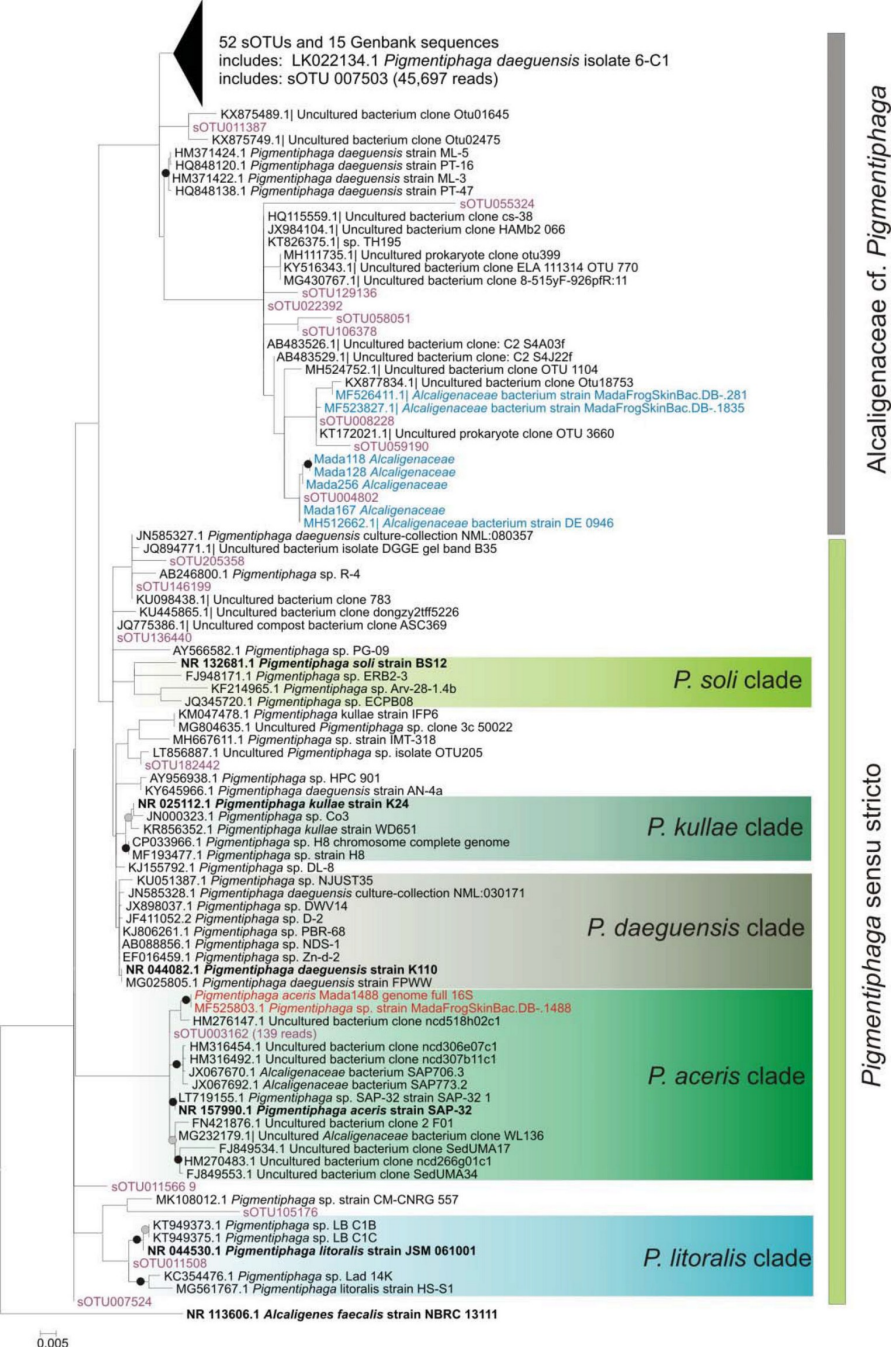
Maximum likelihood phylogenetic tree of selected *Pigmentiphaga* based on DNA sequences of up to 1478 bp of the bacterial 16S rRNA gene. Sequences of amphibian skin bacteria from an Illumina-based amplicon survey[6] are colored purple; isolates from amphibian skin are colored blue. Red color highlights the sequences of the *P*. *aceris* strain used for genome sequencing. Sequences from type or reference strains are boldfaced. *Alcaligenes faecalis*, the type species of the type genus of *Alcaligenaceae*, was used as the outgroup. results of a bootstrap analysis (100 replicates) are marked by gray (bootstrap proportion >50%) and black circles (>70%). Note that due to the inclusion of many short sequences from Illumina amplicon analysis, most nodes did not receive strong bootstrap support.

We aligned short amplicon-based consensus sequences of *Alcaligenaceae* amplicons with the longer 16S sequences of all Madagascan amphibian-derived isolates from a previous work [15] belonging to this family. We then used a series of BLAST and MOLE-BLAST searches, allowing for hits with and without environmental sequences, and restricting searches to type strains or not, to retrieve a representative set of 273 homologous *Alcaligenaceae* sequences of the 16S rRNA gene for analysis of phylogeny and environmental distribution of our focal bacterial taxa. The exploratory analysis of these sequences along with our *Alcaligenaceae* sOTU and isolate sequences placed 92 sOTUs, 8 isolates and 82 related sequences retrieved from GenBank in a clade with the *Pigmentiphaga* type strains. A ML tree calculated on this restricted dataset (Fig 2) reveals a large diversity of *Pigmentiphaga*, many of which are not assignable to any of the described species. A large number of 61 additional sOTUs from the amphibian skin, as well as one isolate from the skin of a fire salamander (DE946; accession number MH512662) and two from Madagascar frogs (Mada281, Mada1835; accession numbers MF526411, MF523827), are placed in a large subclade of putative *Pigmentiphaga* sequences that does not contain any type strain sequences. This subclade also contains sOTU7503, the most widespread *Alcaligenaceae* sOTU in our global amplicon-derived data set (45,697 reads). Various sequences retrieved from GenBank and included in this subclade are named *P*. *daguensis* but are unlikely to belong to this species, given that the type strain is placed in another, phylogenetically distant clade. Whether this diverse subclade is to be assigned to *Pigmentiphaga* definitively, or to another, possibly undescribed genus in the *Alcaligenaceae*, will require additional study.

### Genome characteristics of *Pigmentiphaga aceris* isolated from amphibian skin

One of our isolates (Mada1488) was placed close to *P*. *aceris* and had >99% sequence similarity with the type strain of this species (Table 1). The sequence obtained by direct Sanger sequencing of DNA extracted from this isolate agreed fully with the sequence from both 16S copies found in the assembled genome. One amplicon-derived sOTU also the sequence of this isolate. In our global amphibian data set, 139 of these reads came from the sOTU matching the Mada1488 isolate. This sOTU was found on frogs of the genera *Anaxyrus*, *Boophis*, *Colostethus*, *Craugastor*, *Gephyromantis*, *Eleutherodactylus*, *Lithobates*, *Mantidactylus*, *Mantella*, and *Plethodontohyla*. Thus, the bacterium represented by our culture (Mada1488) was not very common in our Illumina dataset. As it was the only cultured *Pigmentiphaga* from amphibian skin assignable to a known species we nevertheless chose this isolate for genome sequencing, to obtain first data of the genomic background of these bacteria.

**Table 1:** NCBI database matches (97–100% sequence identity) to the 16S rRNA gene of *Pigmentiphaga aceris* (strain Mada1488).

Accession #	Description	Query cover	Percent match	Isolation Source
HM276147.1	Uncultured bacterium clone ncd518h02c1	89%	99.4%	Human skin
NR_157990.1	*Pigmentiphaga aceris* strain SAP-32	94%	99.3%	tree sap
HM270483.1	Uncultured bacterium clone ncd266g01c1	89%	99.2%	human skin
FJ849553.1	Uncultured bacterium clone SedUMA34	95%	99.2%	arctic stream sediment; ultramafic lithology
JX067670.1	Alcaligenaceae bacterium SAP706.3	98%	99.1%	floral nectar
FN421876.1	Uncultured bacterium, clone 2_F01	92%	99.1%	phyllosphere of clover
HM316492.1	Uncultured bacterium clone ncd307b11c1	89%	99.0%	human skin
JX067692.1	Alcaligenaceae bacterium SAP773.2	98%	99.0%	floral nectar
HM316454.1	Uncultured bacterium clone ncd306e07c1	89%	99.0%	human skin
FJ849534.1	Uncultured bacterium clone SedUMA17 1	95%	98.4%	arctic stream sediment; ultramafic lithology
MH667611.1	*Pigmentiphaga* sp. strain IMT-318	94%	97.1%	soil (USA)

In culture, this bacterium forms white, glossy colonies with circular form; the elevation is raised and the margins are entire. In a growth inhibition assay (see [15] for methods) the metabolites produced by this bacterium reduced the growth of the amphibian skin pathogen *Batrachochytrium dendrobatidis* by 60%. BLAST searches in the NCBI nucleotide database revealed that bacterial strains with >97% 16S sequence identity to Mada1488 have been found on human skin, in floral nectar, tree sap, artic stream sediment, and soil (Table 1). The highest identity was found with an uncultured isolate from human skin (99.4% identity), directly followed by the *P*. *aceris* type strain (SAP-32) with 99.3% identity.

**Fig 3:**
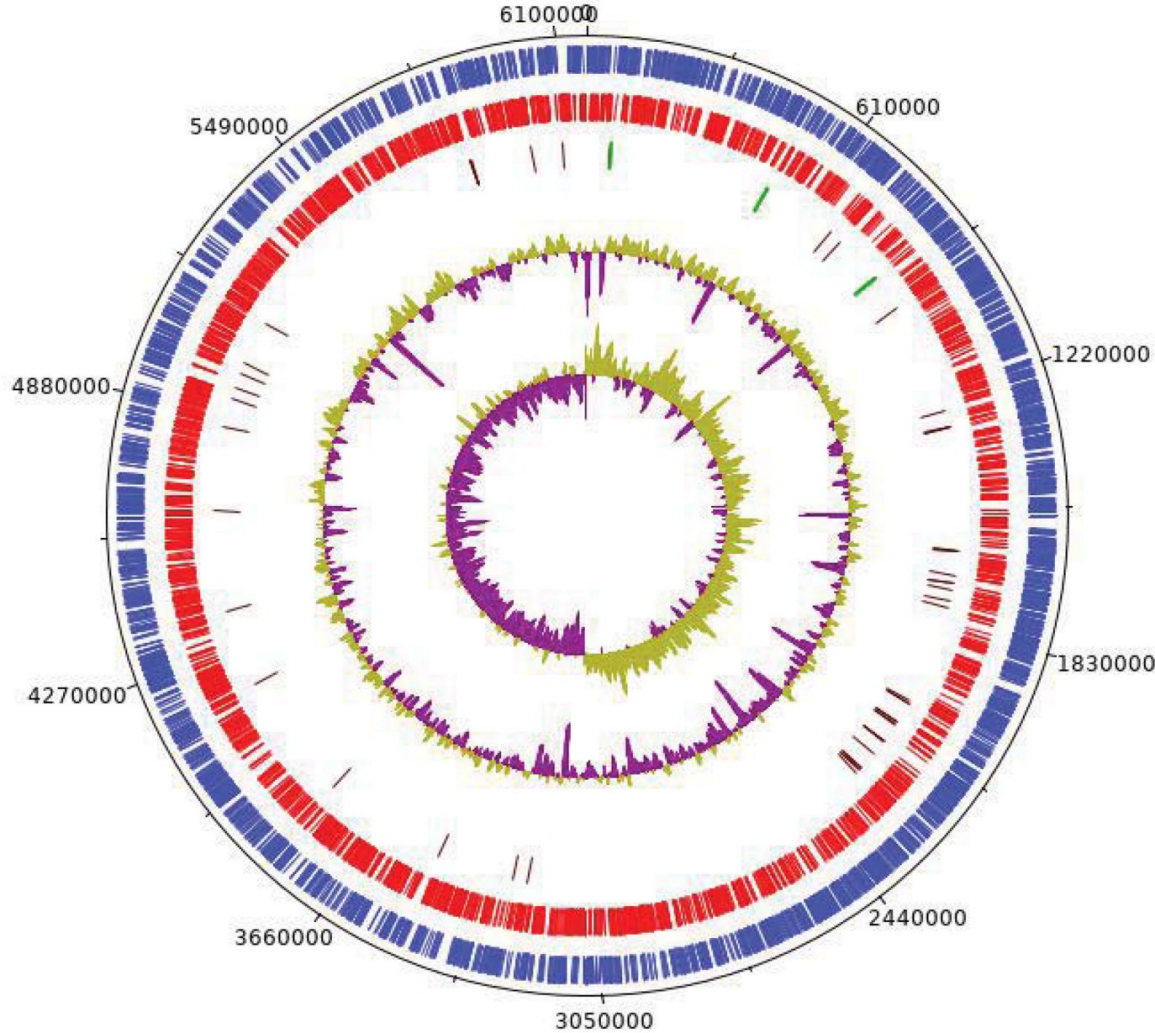
The circular genome of 6,165,255 bp of *Pigmentiphaga aceris* (strain Mada1488). In blue (circle 1) genes lying on the forward strand are shown and in red (circle 2) those on the reverse strand. In circle 3 tRNA genes are shown in brown, often clustered together with green rRNA genes. Circle 4 shows the GC content (G+C)/(A+T+G+C), whereas in circle 5 a GC skew (G−C)/(G+C) is shown. This map has been created using DNAplotter [44].

The complete genome of *Pigmentiphaga aceris* (Mada1488) consists of a single circular chromosome with 6,165,255 bp and a GC content of 62.1%. Prokka predicted 5,300 coding sequences, 57 tRNA genes, and three rRNA operons (Fig 3). A multiple genome alignment suggests that the new genome shows only limited similarities to the five congeneric genomes available (S1 Fig); however, the five available genomes all belong to closely related strains (all >98% 16S similarity to the type strains of *P*. *kullae* and *P*. *daeguensis*, which themselves show 99.6% similarity, questioning the distinctness of these two taxa at the species level).

The newly sequenced genome of *P*. *aceris* (Mada1488) differs in its gene functions compared to other known *Pigmentiphaga* genomes; more specifically, genes associated with environmental information processing and cellular processes were more abundant in Mada1488 (S2 Fig). Natural product biosynthetic gene clusters (BGCs) are moderately represented in the newly sequenced genome, and the cluster content differs from that in the other available *Pigmentiphaga* genomes (S1 Table). Amongst others, *P*. *aceris* (Mada1488) contains a nonribosomal peptide syntethase (NRPS) cluster most likely involved in the production of a peptide siderophore similar to enterobactin [45], and a BGC coding for bacteriocin biosynthetic genes, both missing in the other *Pigmentiphaga*. Bacteriocines are widely occurring, ribosomally produced antimicrobial peptides, presumably with an anti-competitor function [46] and an often narrow activity range against Gram-positive as well as Gram-negative bacteria [47,48]. The most noticeable feature in the comparative *Pigmentiphaga* genomes but absent in *P*. *aceris* (Mada1488) are clusters for β-lactones, a class of protein-inhibiting natural products with a wide range of activities [49]. Furthermore, *P*. *aceris* (Mada1488) lacks genes involved in ectoin biosynthesis. Because ectoins are usually produced to protect the bacteria from environmental extremes like hyperosmotic conditions [50], the lack of ectoins might support this strain being adapted to rather stable environmental conditions.

### Volatile compounds produced by *Pigmentiphaga aceris*

The ability to produce volatile organic compounds (VOCs) is widespread among bacteria [51] and our data demonstrate this ability also in *Pigmentiphaga aceris* (Mada1488). GC/MS analysis of headspace extracts from this bacterium revealed the release of 21 compounds (S1 Table), among them sulfur-containing volatiles such as methanethiole, dimethyl disulfide (**1**), dimethyl trisulfide (**2**), *S*-methyl ethanethioate (**3**), *S*-methyl propanethioate (**4**), *S*-methyl 2-methylpropanethioate (**5**), *S*-methyl 3-methylbutanethioate (**6**) and *S*-methyl phenylethanethioate (**7**), as well as γ-decalactone (**8**) (S3 Fig). Some these compounds, for example, dimethyl trisulfide and *S*-methyl 3-methylbutanethioate, have been associated with inhibition of a variety of plant pathogens [52,53], suggesting they may play a role in suppression of amphibian fungal pathogens, such as *Bd* and *Bsal*.

## Conclusions

To our knowledge, *Pigmentiphaga aceris* (Mada1488) is the first amphibian skin-derived bacterial isolate with a full genome sequenced. Overall, the genome does not present any outstanding characteristics, in line with the hypothesis that the amphibian cutaneous microbiome mainly consists of generalist species recruited from environmental reservoirs. It is remarkable that bacterial strains most similar to Mada1488 have multiple times been found in plant-associated microbiomes (tree sap, nectar, phyllosphere). The finding of a *Pigmentiphaga* similar to the strain described herein on human skin may be explained by a bias of information in genetic databases towards human-derived microbes, but also confirms that these bacteria are not strictly associated to plants only. Yet, it is tempting to relate the apparent common occurrence of *Pigmentiphaga* on plants to its high abundance in treefrogs which may have acquired it from plant-associated reservoirs. To test this hypothesis, in-depth analysis of additional bacteria differentially abundant on arboreal vs. terrestrial amphibians may be rewarding. A wider sampling of genomes represented in amphibian cutaneous microbiomes will be a crucial step to better understand functional properties of these bacterial communities and their potential role in defense against pathogens.

### Data availability

The complete genome sequence of *Pigmentiphaga aceris* Mada1488 has been deposited at NCBI GenBank under the accession no. CP043046. The version described in this paper is the first version. (BioProject no. PRJNA561098). The 16S sequence of this isolate is archived under accession no. MF525803.1. Accession numbers of sequences used in the phylogenetic analysis are given in the respective tree. Accession numbers for the raw data of amplicon analyses are summarized in a previous study [6].

## Supporting information

S1 Fig **Comparison of genomic composition of the *Pigmentiphaga aceris* strain isolated from amphibian skin** (A) to the five other *Pigmentiphaga* genomes available: (A) *Pigmentiphaga* sp. H8, (B) *P*. sp. NML030171, (C) *P*. sp. NML080357, (D) *P*. *kullae* K24, (E) *P*. sp. IMT-318. The figure shows a multiple genome alignment calculated with Mauve (Darling et al. 2004), using A as reference. Colinear blocks are indicated by identical colors and indicate homologous DNA regions shared by two or more genomes without sequence rearrangements, and are indicated below the black horizontal line if representing reverse complements of the respective sequence of the reference. Note similarities between genomes A-C, larger differences of D and E, and massive differences in the arrangement of the newly sequenced *P*. *aceris* genome (F).
(JPG)

S2 Fig Comparative summary of gene function across the newly sequenced *Pigmentiphaga aceris* (red box) and other available genomes from this genus. Pie charts were created directly from BlastKOALA. Colors for a given functional categories are consistent across each chart; categories are ordered by abundance within a given pie chart.
(PDF)

S3 Fig **Selected volatile compounds released by *Pigmentiphaga aceris* (Mada1488):** methanethiole, dimethyl disulfide (1), dimethyl trisulfide (2), S-methyl ethanethioate (3), S-methyl propanethioate (4), S-methyl 2-methylpropanethioate (5), S-methyl 3-methylbutanethioate (6) and S-methyl phenylethanethioate (7), as well as γ-decalactone (8).
(PDF)

S1 Table Natural product biosynthetic gene clusters (BGCs) in available *Pigmentiphaga* genomes as predicted by AntiSMASH. (DOCX)

S2 Table Volatile compounds released by *Pigmentiphaga aceris* (Mada1488). Numbered compounds 1–8 are those shown in S2 Fig.
(DOCX)
