# Supplementary figures and images for: Amphibian skin-associated Pigmentiphaga: Genome sequence and occurrence across geography and hosts
Source: PLoS One. 2019 Oct 11;14(10):e0223747. doi: 10.1371/journal.pone.0223747 (PMC6788695; doi:10.1371/journal.pone.0223747)

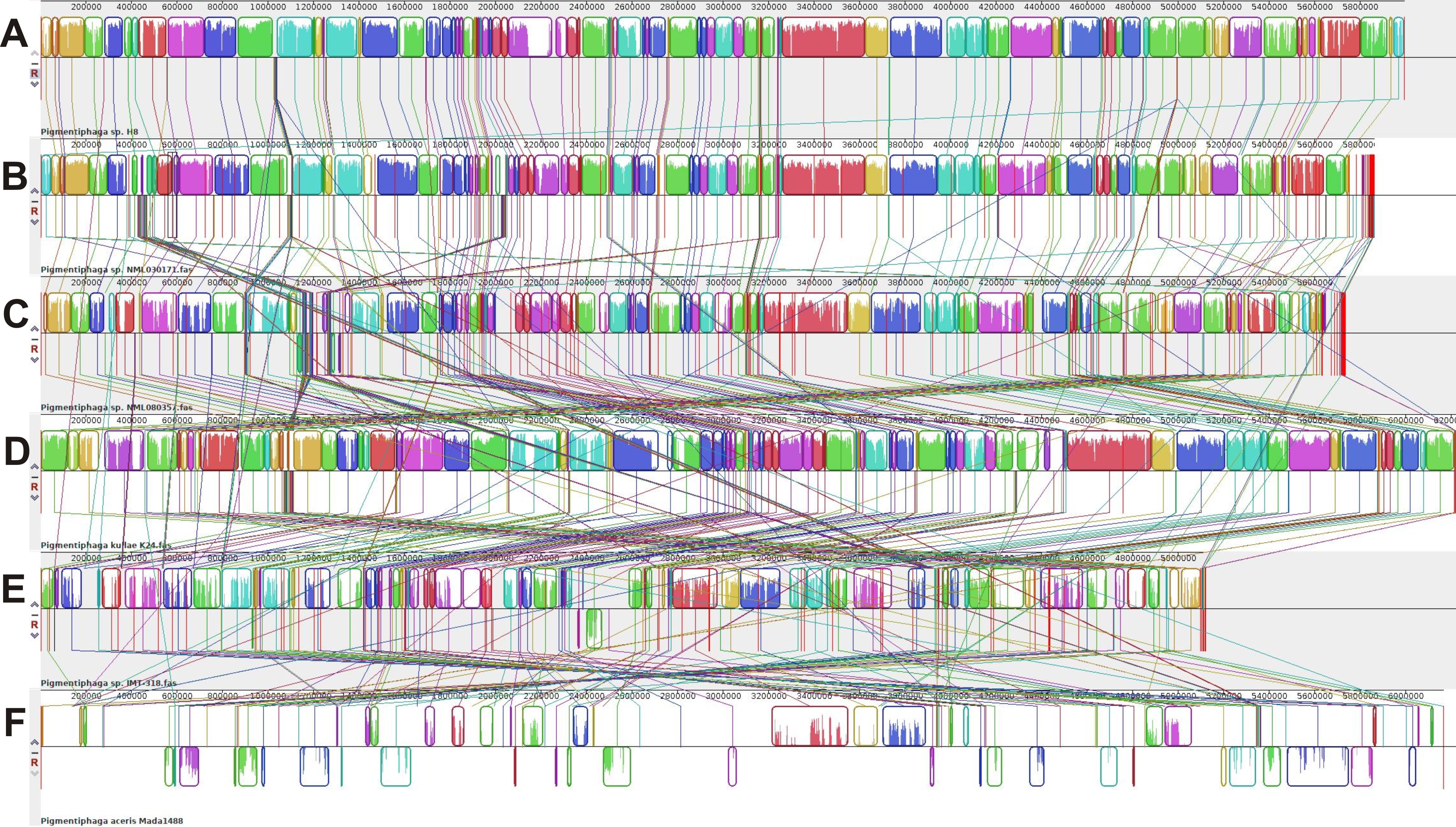

Supplement: S1 Fig — Comparison of genomic composition of the Pigmentiphaga aceris strain isolated from amphibian skin (A) to the five other Pigmentiphaga genomes available: (A) Pigmentiphaga sp. H8, (B) P. sp. NML030171, (C) P. sp. NML080357, (D) P. kullae K24, (E) P. sp. IMT-318. The figure shows a multiple genome alignment calculated with Mauve (Darling et al. 2004), using A as reference. Colinear blocks are indicated by identical colors and indicate homologous DNA regions shared by two or more genomes without sequence rearrangements, and are indicated below the black horizontal line if representing reverse complements of the respective sequence of the reference. Note similarities between genomes A-C, larger differences of D and E, and massive differences in the arrangement of the newly sequenced P. aceris genome (F). (JPG) [file pone.0223747.s001.jpg]

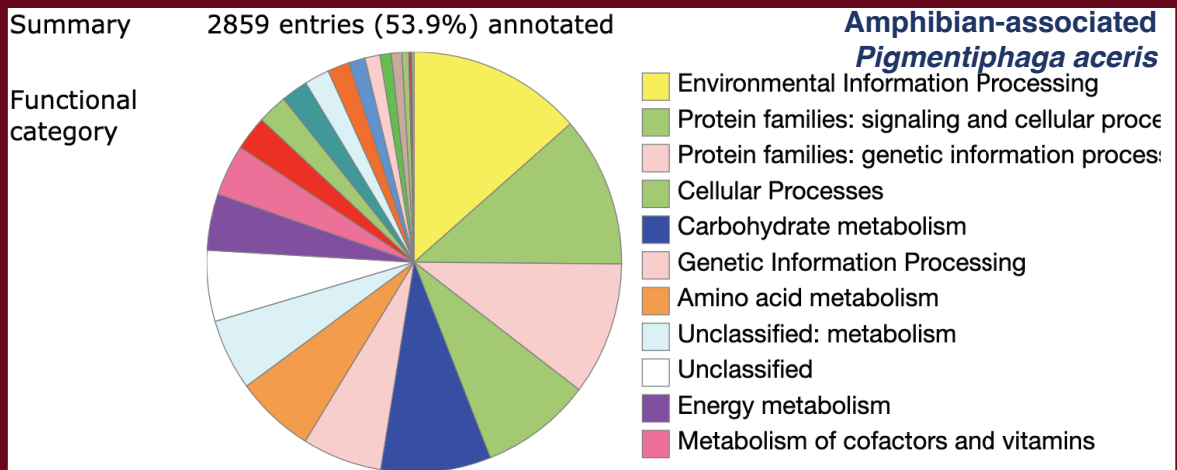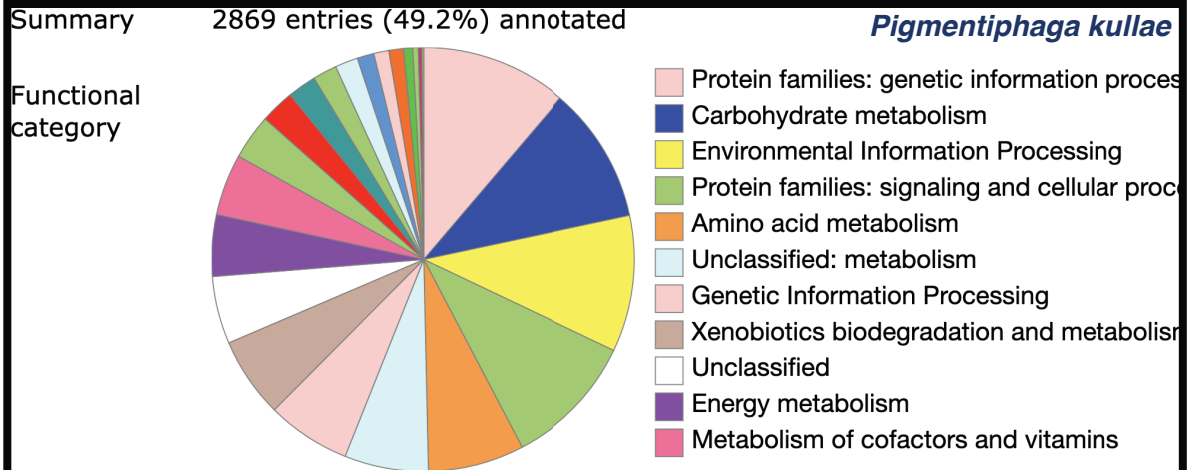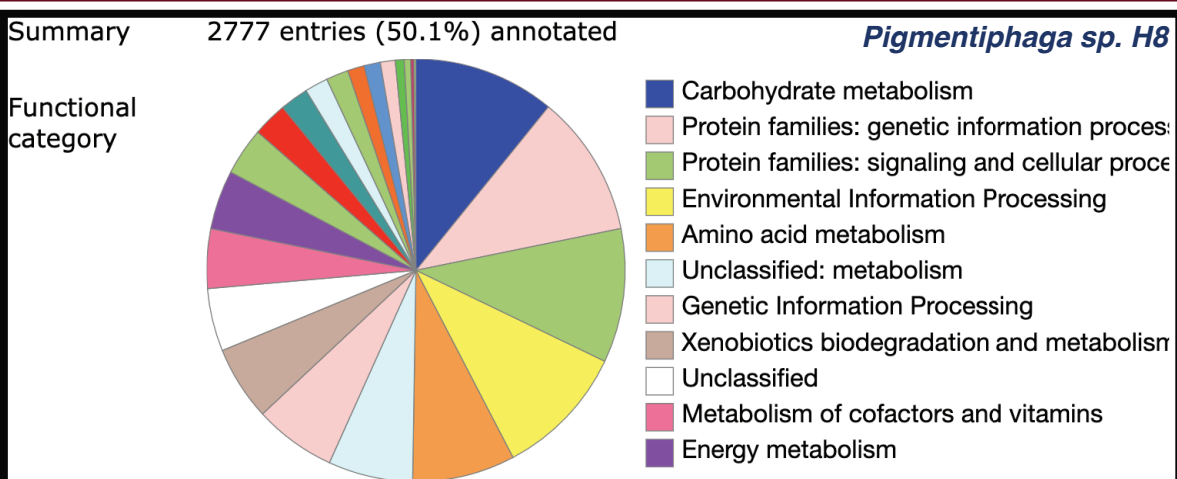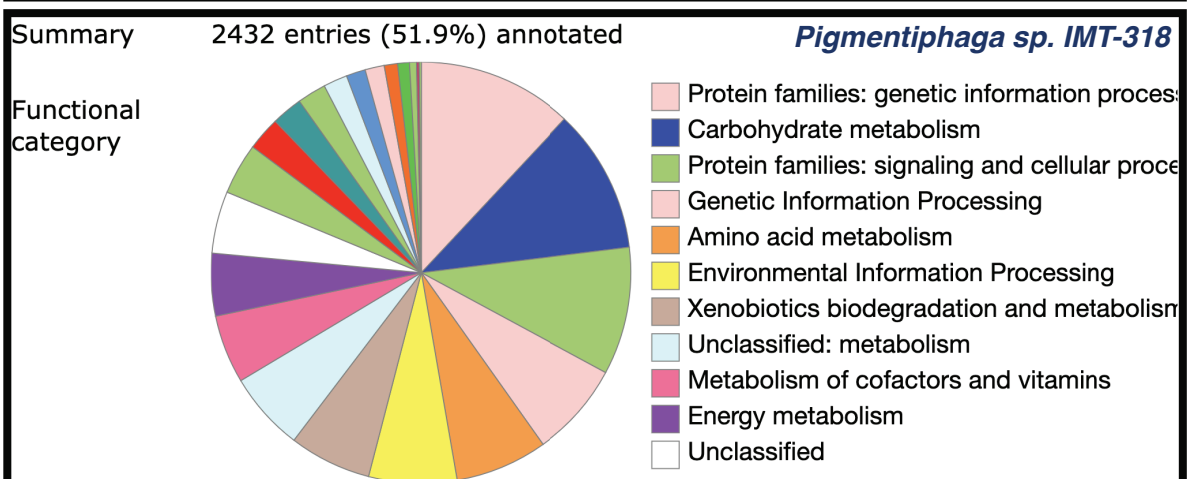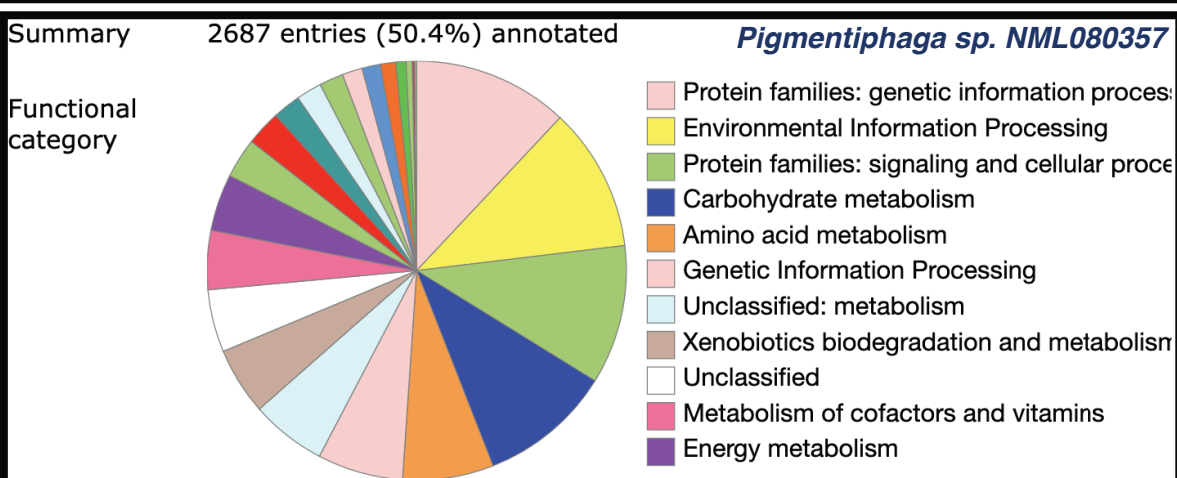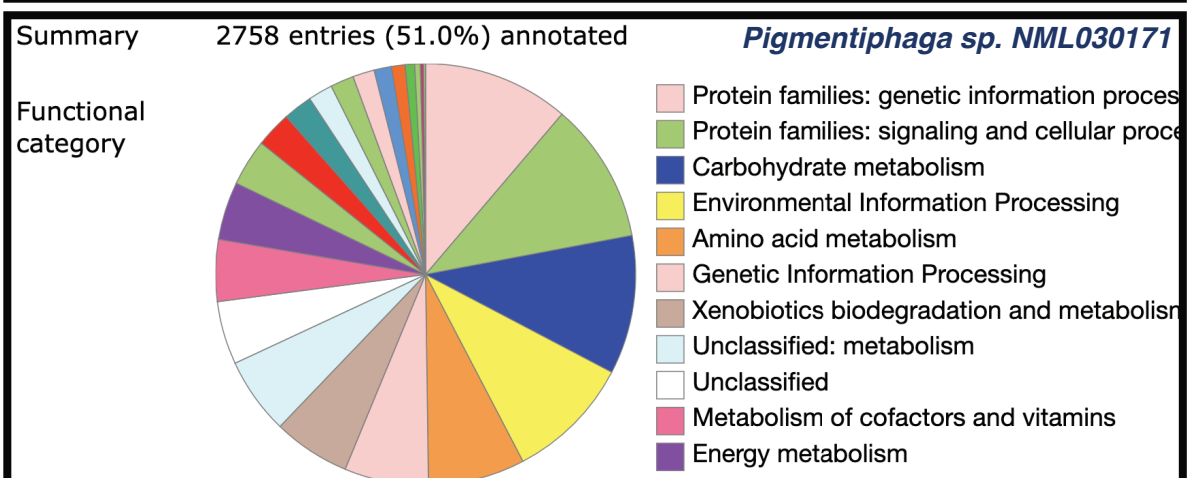

Supplement: S2 Fig — Pie charts were created directly from BlastKOALA. Colors for a given functional categories are consistent across each chart; categories are ordered by abundance within a given pie chart. (PDF) [file pone.0223747.s002.pdf]

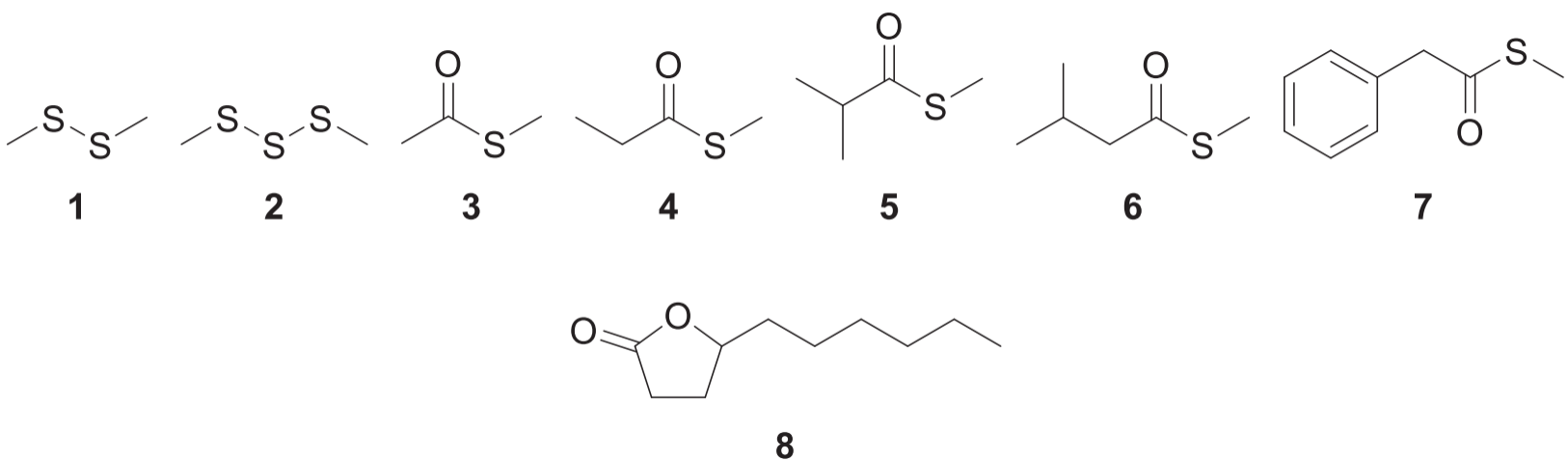

Supplement: S3 Fig — Selected volatile compounds released by Pigmentiphaga aceris (Mada1488): methanethiole, dimethyl disulfide (1), dimethyl trisulfide (2), S-methyl ethanethioate (3), S-methyl propanethioate (4), S-methyl 2-methylpropanethioate (5), S-methyl 3-methylbutanethioate (6) and S-methyl phenylethanethioate (7), as well as γ-decalactone (8). (PDF) [file pone.0223747.s003.pdf]
