# Supplementary material for: Amphibian skin-associated Pigmentiphaga: Genome sequence and occurrence across geography and hosts
Source: PLoS One. 2019 Oct 11;14(10):e0223747. doi: 10.1371/journal.pone.0223747 (PMC6788695; doi:10.1371/journal.pone.0223747)
Supplement: S1 Table — (DOCX) [file pone.0223747.s004.docx]

**SUPPLEMENTARY MATERIALS**

**Amphibian skin-associated *Pigmentiphaga*: genome sequence and occurrence across geography and hosts**

Molly C. Bletz^1,7,*^, Boyke Bunk^2^, Catherine Spröer^2^, Peter Biwer^3^, Silke Reiter^4^, Falitiana C. E. Rabemananjara^5^, Stefan Schulz^3^, Jörg Overmann^2,6^, Miguel Vences^7^

**Table S1.** Natural product biosynthetic gene clusters (BGCs) in available *Pigmentiphaga* genomes as predicted by AntiSMASH.

|  | **Studied genome** | | | | | |
| --- | --- | --- | --- | --- | --- | --- |
| **Natural product class** | Mada 1488 | GCA_900606115.1 | GCA_003854895.1 | GCA_004216695.1 | GCA_002188465.1 | GCA_002188635.1 |
| NRPS | 1 |  |  |  |  |  |
| Terpen | 1 | 1 | 1 | 1 | 1 | 1 |
| Bacteriocin | 1 |  |  |  |  |  |
| Arylepolyene | 1 | 1 |  |  |  |  |
| Resorchinol | 1 |  | 1 | 1 |  | 1 |
| Lassopeptide |  | 1 |  |  |  |  |
| Ectoin |  | 1 | 1 | 1 | 1 | 1 |
| Betalactone |  |  | 2 | 2 | 2 | 3 |
