# Supplementary material for: Amphibian skin-associated Pigmentiphaga: Genome sequence and occurrence across geography and hosts
Source: PLoS One. 2019 Oct 11;14(10):e0223747. doi: 10.1371/journal.pone.0223747 (PMC6788695; doi:10.1371/journal.pone.0223747)
Supplement: S2 Table — Numbered compounds 1–8 are those shown in S2 Fig. (DOCX) [file pone.0223747.s005.docx]

**SUPPLEMENTARY MATERIALS**

**Amphibian skin-associated *Pigmentiphaga*: genome sequence and occurrence across geography and hosts**

Molly C. Bletz^1,7,*^, Boyke Bunk^2^, Catherine Spröer^2^, Peter Biwer^3^, Silke Reiter^4^, Falitiana C. E. Rabemananjara^5^, Stefan Schulz^3^, Jörg Overmann^2,6^, Miguel Vences^7^

**Table S2.** Volatile compounds released by *Pigmentiphaga aceris* (Mada1488). Numbered compounds 1-8 are those shown in Supplementary Fig. S2.

| **No.** | **Compounds** |
| --- | --- |
| **1** | Dimethyl disulfide |
| **2** | Dimethyl trisulfide |
| **3** | *S*-Methyl ethanethioate |
| **4** | *S*-Methyl propanethioate |
| **5** | *S*-Methyl 2-methylpropanethioate |
| **6** | *S*-Methyl 3-methylbutanethioate |
| **7** | *S*‑Methyl phenylethanethioate |
| **8** | γ-Decalactone |
|  | Methanethiol |
|  | Acetic acid |
|  | 3-Methyl-1-butanol |
|  | Heptanal |
|  | Phenol |
|  | Octanal |
|  | Phenylacetaldehyde |
|  | 2-Nonanone |
|  | 2-Phenylethanol |
|  | Acetophenone |
|  | 4-Methylquinoline |
|  | 2-Tridecanone |
|  | 2-Pentadecanone |
